# Disruption of the microtubule network alters cellulose deposition and causes major changes in pectin distribution in the cell wall of the green alga, *Penium margaritaceum*

**DOI:** 10.1093/jxb/ert390

**Published:** 2013-11-27

**Authors:** David S. Domozych, Iben Sørensen, Carly Sacks, Hannah Brechka, Amanda Andreas, Jonatan U. Fangel, Jocelyn K. C. Rose, William G. T. Willats, Zoë A. Popper

**Affiliations:** ^1^Department of Biology and Skidmore Microscopy Imaging Center, Skidmore College, Saratoga Springs, NY 12866, USA; ^2^Department of Plant Biology, Cornell University, Ithaca, NY 14853, USA; ^3^Department of Plant and Environmental Sciences, University of Copenhagen, Faculty of Science, Thorvaldsensvej 40, 1871 Frederiksberg, Denmark; ^4^Botany and Plant Science, School of Natural Sciences and Ryan Institute for Environmental, Marine and Energy Research, National University of Ireland, Galway, Ireland

**Keywords:** Cellulose, homogalacturonan, microtubule, oryzalin, pectin, *Penium*.

## Abstract

Application of the dintroaniline compound, oryzalin, which inhibits microtubule formation, to the unicellular green alga *Penium margaritaceum* caused major perturbations to its cell morphology, such as swelling at the wall expansion zone in the central isthmus region. Cell wall structure was also notably altered, including a thinning of the inner cellulosic wall layer and a major disruption of the homogalacturonan (HG)-rich outer wall layer lattice. Polysaccharide microarray analysis indicated that the oryzalin treatment resulted in an increase in HG abundance in treated cells but a decrease in other cell wall components, specifically the pectin rhamnogalacturonan I (RG-I) and arabinogalactan proteins (AGPs). The ring of microtubules that characterizes the cortical area of the cell isthmus zone was significantly disrupted by oryzalin, as was the extensive peripheral network of actin microfilaments. It is proposed that the disruption of the microtubule network altered cellulose production, the main load-bearing component of the cell wall, which in turn affected the incorporation of HG in the two outer wall layers, suggesting coordinated mechanisms of wall polymer deposition.

## Introduction

Plant cell walls are composites of polymers that are assembled and organized into intricate structures that surround the protoplast, where they serve multiple roles including defence, turgor resistance and controlled cell growth, water and mineral uptake, and communication (Baskin *et al.*, 2004; Cosgrove, 2005; Sarkar *et al.*, 2009; Keegstra, 2010; Fry, 2011). Cell wall architecture is highly dynamic, and synthesis, assembly, and any subsequent remodelling require precisely coordinated interactions between the cell endomembrane system, cytoskeletal network, plasma membrane, and multiple cross-talking signal transduction pathways. Cell wall production and maintenance therefore involve not just a substantial amount of the total photosynthate, but also a major portion of the genetic repertoire (Popper *et al.*, 2011).

The structural and developmental characteristics and functional competency of the plant wall are also fundamentally affected by complex multipolymeric associations. The nature of these interactions, especially during development and in response to environmental stresses, is poorly understood and only recently has this been the focal point of detailed study. For example, cellulose microfibrils are generally described as being tethered by xyloglucan and other hemicellulosic (cross-linking glycan) polymers, and these have been proposed to influence microfibril slippage during wall and cell expansion (Popper and Fry, 2005, 2008; Fry, 2011); the nature, extent, and significance of this cross-linking have recently been discussed (Cosgrove and Jarvis, 2012; Park and Cosgrove, 2012). There is also recent evidence that the neutral sugar side chains (e.g. arabinans and galactans) of the pectin class rhamnogalacturonan-I (RG-I) may be directly bound to cellulose (Zykwinska *et al*., 2005, 2007). Yoneda *et al.* (2010) further suggested that pectin cross-bridges support and maintain the direction of cellulose microfibril orientation and slippage during cell expansion. However, there are doubtless many other interpolymeric associations that are critical for wall architecture and function, but that have yet to be recognized and characterized.

Evaluating such interactions within the context of multicellular plants is very challenging, and the extraction of cell wall polymeric complexes inevitably disrupts or abolishes a number of the molecular associations. Moreover, the physical restriction of specific polymer probes in dense tissues and the inability to use live material in many labelling and analytical protocols effectively further limit dissection of interpolymeric interactions. In contrast, the identification and use of a unicellular plant system, particularly one with clearly defined cell wall polymer domains, would significantly enhance such studies.

A unicellular taxon of the Charophycean green algae (CGA or Streptophyta; i.e. the group of green algae most closely related to land plants; Lewis and McCourt, 2004; Wodniok *et al.*, 2011), *Penium margaritaceum*, has a number of characteristics that suggest it would provide a potentially valuable model system for the study of cell wall development, including interpolymeric associations. First, *Penium* only produces a permanent primary cell wall, comprising two prominent polymeric domains that are easily identified by microscopy: a pectic domain primarily consisting of homogalacturonan (HG) organized into a lattice-like network in the outer layer of the wall; and an inner domain consisting mostly of cellulose, together with smaller amounts of other glycan classes (Sørensen *et al.*, 2010, 2011; Domozych *et al.*, 2011). Secondly, the focal point of HG secretion, which in *Penium* appears to drive cell wall growth and cell development, is a clearly defined narrow band located at the cell centre or isthmus, or the isthmus band (Domozych *et al.*, 2009*b*). This facilitates visualization of wall polymer secretion in a spatially well-defined area. Thirdly, *Penium* can be grown in large, fast-growing cultures, enabling extraction of substantial amounts of cell wall material for biochemical and immuno-based screening (Møller *et al*., 2007; Sørensen and Willats, 2011). Fourthly, wall polymer dynamics can be conveniently monitored by live cell labelling utilizing probes such as monoclonal antibodies (mAbs) directed against higher plant wall polymers or carbohydrate-binding modules (CBMs; Domozych *et al.*, 2011). Finally, the cell cultures can be readily treated with agents that promote or disrupt cellular processes, including enzymes and pharmacological inhibitors, at precise concentrations and over controlled time periods.

In this study, the structural and developmental dynamics of the pectin and cellulose domains during *Penium* cell wall expansion and cell morphogenesis following treatment with the dinitroaniline herbicide, oryzalin, were analysed. This compound blocks microtubule polymerization and consequently inhibits cell wall development and anisotropic growth (Hugdahl and Morejohn, 1993). A combination of high resolution microscopy, polysaccharide microarray analysis, and experimental manipulation was used to study oryzalin-induced changes to the cell wall. Distinct effects of oryzalin on the pectin and cellulose domains of the cell wall and concurrent alterations to the cytoskeletal system are described, and the implications of the results for the control and coordination of cell wall disassembly are discussed.

## Materials and methods

### General

*Penium margaritaceum* (‘Skd-8’ clone, Skidmore College Algal Culture Collection) was grown in liquid Woods Hole medium (WHM; Domozych *et al.*, 2007) under the following conditions: 5400 lux of cool white fluorescent light, 18±1 °C, 16h light/8h dark photocycle. Subcultures were made every 2 weeks and cells used for experiments were collected after 5–7 d in culture. Cells were harvested and washed as previously described (Domozych *et al.*, 2007).

Oryzalin was obtained from AccuStandard (New Haven, CT, USA) and the final concentration chosen for experimental procedures was 280nM as this concentration provided the most evident phenotypes. Specific experiments were conducted in 5ml aliquots of culture medium, each containing 500 cells ml^–1^. After the addition of oryzalin (from a stock solubilized in methanol), the cells were cultured under the conditions described above. Control experiments included growing cells in 0.01% methanol. Reversibility experiments entailed harvesting oryzalin-treated cells at various time intervals, washing five times in fresh WHM, and culturing in fresh WHM. Washed cells were then monitored via microscopy over the next 72h. Total reversibility of effects could be visualized in cells incubated in oryzalin for ≤96h. Cells were also treated with isoxaben (10 μM) or dichloronitrobenzile (DCB; 0.2 μM; Sigma Chemical, St Louis, MO, USA) and monitored after 24h.

### Live cell labelling

Treated and untreated cells were harvested, washed with WHM, and labelled with the following mAbs, as previously described (Domozych *et al.*, 2007): JIM5 [specificity for HG with a relatively low degree of esterification (DE); Clausen *et al.*, 2003]; JIM7 (specificity for relatively high DE HG; Clausen *et al.*, 2003), and INRA-RU2 [specificity for (1→2)-α-l-rhamnose (Rha) (1→4)-α-d-galacturonic acid (GalA) p-(1→7) with at least two Rha–GalA acid repeats; Ralet *et al.*, 2010]. All primary antibodies were obtained from Plant Probes (Leeds, UK), with the exception of INRA-RU2, which was a generous gift from Dr M.-C. Ralet (INRA Nantes, France). Secondary antibodies for immunofluorescnce studies included anti-rat or anti-mouse antibodies conjugated with tetramethylrhodamine isothiocyanate (TRITC) or fluorescein isothiocyanate (FITC) (Sigma). For labelling with CBM3a (specificity for crystalline cellulose), the protocols recommended by the supplier (Plant Probes; also see Blake *et al.*, 2006) were employed with the modification that WHM was used as the labelling buffer. Labelled cells were either viewed via light microscopy (LM) or washed with WHM and placed back into culture. Aliquots of cells were subsequently removed at various time intervals and viewed via fluorescence light microscopy (FLM) either on an Olympus BX-60 LM (NY-NJ Scientific, New Jersey, USA) equipped with fluorescence optics, or an Olympus BX-61 LM equipped with a Fluoview 300 confocal laser scanning microscopy (CLSM) system. In order to ascertain mAb labelling patterns relative to the chloroplast, which fills most of the protoplast, some mAb-labelled cells were initially imaged to assess wall labelling and then with the argon laser which provided the autofluorescence exhibited by the chloroplast in the background. The image stacks from each were superimposed to yield dual-labelled images. Similarly, some cells were first labelled with JIM5, placed back in culture, and then labelled with JIM7. For general morphological studies, cells were observed with differential interference contrast light microscopy (DIC-LM).

### Enzyme pre-treatment

Aliquots of washed cells were treated for 24h or 48h with pectate lyase (PL) (Megazyme, IR; E-PECLY, final concentration 1.2U) or cellulase (Sigma Chemical; #0615; 500 μg ml^–1^). The cells were then collected and resuspended in 280nM oryzalin in either the PL or cellulase solutions for 24–48h. Cells were collected and viewed with DIC-LM, or labelled with JIM5 and observed with FLM or CLSM.

### Microtubule and actin labelling

Immunolocalization of microtubules was performed using the freeze shatter technique of Wasteneys *et al.* (1997). Rhodamine–phalloidin labelling was performed using the method described by Holzinger *et al.* (2002).

### Quantitative measurements

The surface area (SA) of a cell covered by new cell wall, as recognized by new HG in relation to whole cell SA, was calculated for JIM5-labelled cells incubated in oryzalin for 48h or 72h, or in control cultures. The cylindrical morphology of *Penium* and the constant cell width (17 μm) of each cell allows for SA measurements to be obtained using the standard formula for determining the SA of a cylinder: SA=2 (π×r^2^)+(2π×r)×L, where r=radius of the cell, L=length of the designated area (i.e. length of the cell or length of the cell area with newly deposited HG). For L, the length of specific areas with new cell wall was calculated as the non-fluorescent zones produced post-initial JIM5 labelling. Measurements were made using standard Cell B software (Olympus). Triplicate samples of 100 cells each were measured and a 0.98 (SA) curvature factor employed to account for the blunt rounding of the cells at the poles. For calculating SA of the swollen, spherical isthmus regions of oryzalin-treated cells, the diameter of the central, spherical, swollen zones was measured, in addition to the adjacent cylindrical polar regions. The SA of the spherical regions was determined using the standard formula for a sphere: SA=4πr^2^, where, r=the radius of the sphere. This SA was added to the surface areas of the cylindrical regions at the poles to determine whole cell SA of treated cells.

### Polysaccharide microarray analysis

Polysaccharide microarray analysis was performed as described by Møller *et al*. (2007). Supernatants of extracted cell wall material were spotted in three replicates and three dilutions, and three independent analyses were carried out. Mean spot signals from the three experiments are presented as a heatmap created using the online tool available at http://bar.utoronto.ca/ntools/cgi-bin/ntools_heatmapper.cgi, with the values normalized to the highest value (set to equal 100). A cut-off of 5% of the highest mean signal value was imposed and values below this are represented as 0. Antibodies and CBM3a were obtained from PlantProbes, CCRC (University of Georgia, Athens, GA, USA), Dr M.-C. Ralet (INRA, Nantes, France), or BioSupplies (Parkville, Australia).

### Transmission electron microscopy (TEM)

For TEM analyses, cells were harvested, washed, and spray frozen in liquid propane cooled with liquid nitrogen (see Domozych *et al.*, 2009*b*). Samples were then freeze substituted at –80 °C for 72h in 0.5% glutaraldehyde/1% osmium tetroxide (EMS, Ft. Washington, PA, USA). The samples were then slowly warmed to room temperature over 8h, washed with acetone, and infiltrated/embedded between two plastic Aclar (EMS) sheets in Spurrs low viscosity epoxy plastic (EMS). After polymerization of the plastic, the Aclar was removed, and individual cells were selected, excised from the thin plastic sheet with a razor blade, and mounted with super glue onto a blank plastic mould. Sections (60–80nm) were cut on a Reichert Ultracut ultramicrotome (MOC, Valley Cottage, NY, USA), stained with conventional uranyl acetate/lead citrate, and viewed with either a JEOL 1010 transmission electron microscope (Peabody, MA, USA) or a Zeiss Libra 120 transmission electron microscope (Peabody). In order to enhance HG imaging, some cells were treated prior to fixation with the chelator cyclohexanediaminetetraacetic acid (CDTA; 2h; room temperature). For immunogold labelling, the protocol of Domozych *et al.* (2009*a*) was employed. For enhancement of general ultrastructural and immunogold labelling, some sections were analysed with darkfield optics.

### Variable pressure scanning electron microscopy (VPSEM)

Cells were harvested, washed, and 100 μl aliquots of dense cell suspensions from the pellet were placed on circular 0.8cm diameter nitrocellulose sheets. The cells were allowed to settle on the membrane and excess growth medium was removed with filter paper. Each sheet was plunge-frozen in liquid nitrogen and then placed on a JEOL cryostub, which had been pre-cooled with liquid nitrogen. Cells were viewed on a JEOL 6480 variable pressure scanning electron microscope under the following conditions: 30 Pa, 10kV, and 60 spot size.

### Field emission scanning electron microscopy (FESEM)

Harvested and washed cells were frozen in liquid nitrogen, freeze dried, and placed on stubs coated with double-sided sticky tape. Cells were sputter coated with gold/palladium and imaged using a Zeiss Neon-40 EsB FIB-B scanning electron microscope.

## Results

### Morphology and immunolabelling patterns of cultured *Penium*

**Fig. 1.**
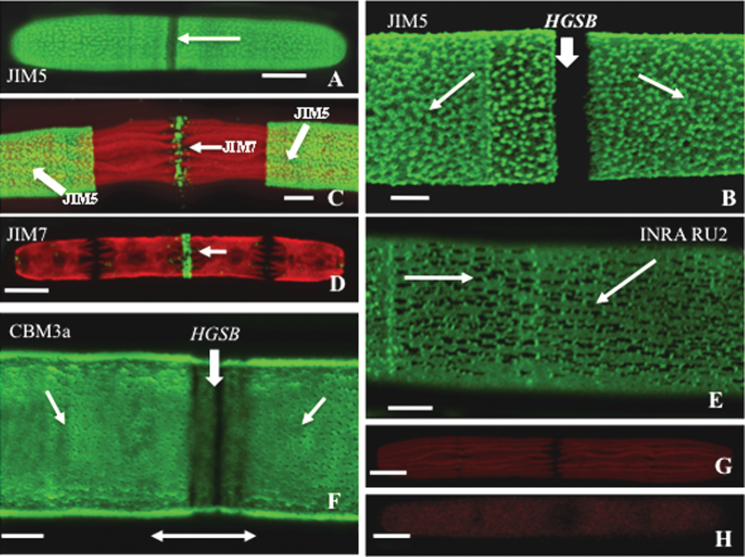
Wall polymer labelling of *Penium*. (A) JIM5 (specificity toward relatively low esterified HG) labels the HG of the outer wall layer. The unlabelled band (arrow) corresponds to the isthmus zone (HG secretion band; isthmus band). Scale bar=11 μm. (B) Magnified view of the JIM5-labelled lattice (arrows) surrounding the isthmus band. Scale bar=3.0 μm. (C) JIM7 (specificity toward relatively highly esterified HG) labelling (small arrow) at the isthmus band underlined by the autofluorescing multilobed chloroplasts. JIM5 labelling highlights the older wall regions (large arrows). Scale bar=5 μm. (D) JIM7 labelling of the isthmus band (arrow) without co-labelling with JIM5. Scale bar=12 μm. (E) RG-I labelling with the mAb INRA-RU2 (specificity toward the backbone of RG-I). The unlabelled puncta (arrows) most probably represent shadows of HG of the outer layer. Scale bar=3 μm. (F) CBM3a (specificity toward crystalline cellulose) labelling of the cellulosic layer (small arrows) revealing the thin unlabelled isthmus band at the isthmus (arrow). Scale bar=3.0 μm. (G) mAb control labelling where primary antibody was eliminated from the labelling process. Scale bar=12 μm. (H) CBM3a control where the CBM3a was left out of the labelling process. Scale bar=12 μm. All images were taken using CLSM.

**Fig. 2.**
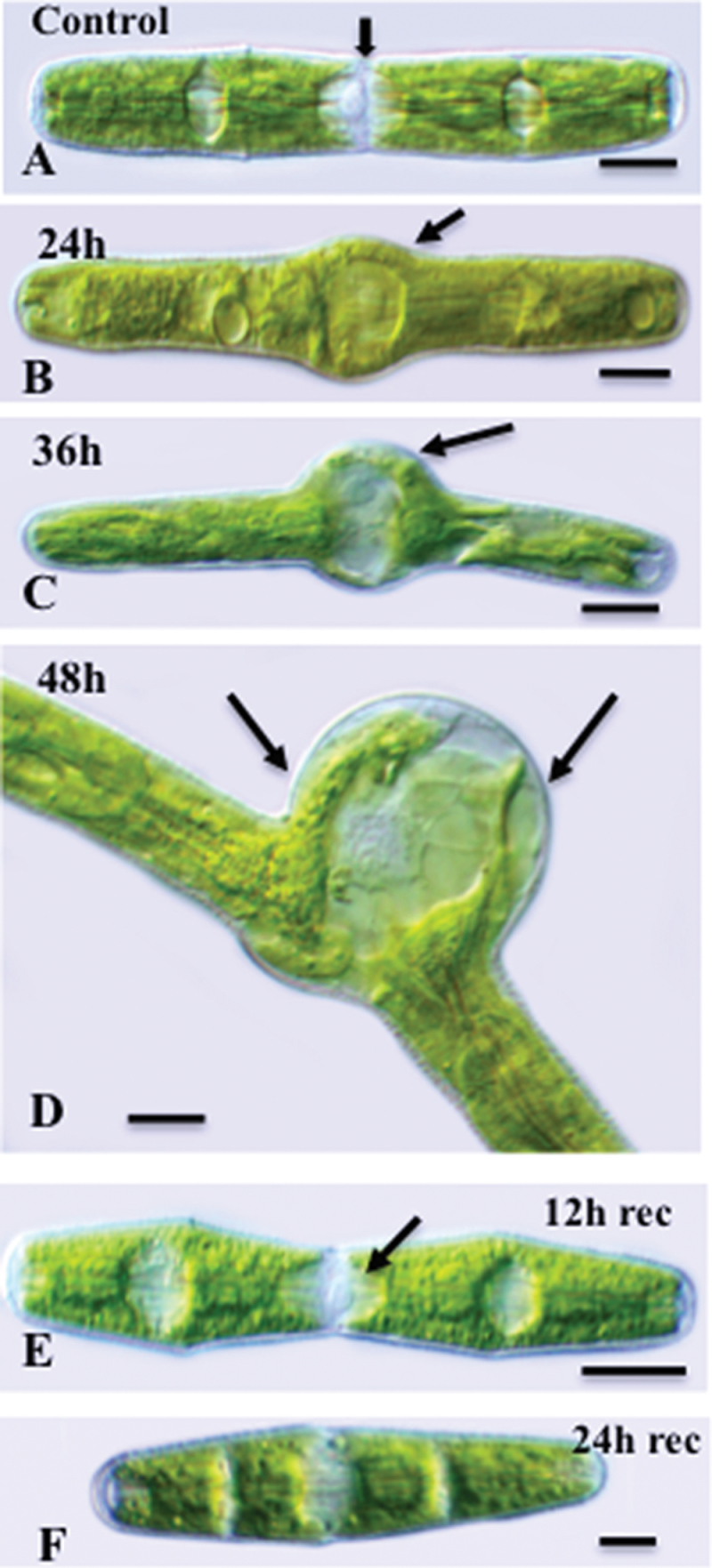
Effects of oryzalin on cell morphology. (A) Typical cell morphology of an untreated cell demonstrating the two semi-cells joined together at the central isthmus (arrow). Scale bar=15 μm. (B) After 24h of oryzalin treatment, a noticeable swelling (arrow) of the isthmus was observed. Scale bar=15 μm. (C). After 36h of treatment, the swelling increased dramatically. Scale bar=14 μm. (D). After 48h of treament, portions of the chloroplasts (arrows) filled the lumen of the swelling and surrounded the nucleus. Scale bar=14.5 μm. (E) After 12h of recovery, the cell returned to its typical cylindrical morphology. This is seen with a narrowing of the cell at the isthmus band (arrow). Two older swollen regions remaining from oryzalin treatment flank the isthmus. Scale bar=14 μm. All images were taken using DIC.

Under normal growth conditions, *P. margaritaceum* is an elongate cylinder with rounded poles. Each cell is ~17 μm wide and cell length varies from 150 μm to 220 μm (Figs 1A, 2A). Live cells may be labelled with mAbs with specificity for epitopes present in land plant cell wall polymers (see also Domozych *et al.*, 2011) or CBMs, and placed back into growth medium where they continue division/expansion and retain the label for 10 d or more, depending on the polymer in question. JIM5, an mAb that recognizes HGs with a relatively low DE, labels a lattice-like structure over most of the cell surface (Fig. 1A; Domozych *et al.*, 2011) except for a narrow, non-labelled band in the isthmus region (Fig. 1B). This band represents the major HG secretion zone during pre-division (i.e. the isthmus band; or ‘HGSB’, Domozych *et al.*, 2009*b*) and is labelled by the mAb JIM7, which recognizes HG with relatively high DE (Fig. 1C, D). RG-I was also identified in the cell wall using INRA-RU2 (Fig. 1E) but, unlike JIM5, INRA-RU2 localized in a layer below the outer wall lattice and in a more homogenous labelling that was interrupted by dark puncta. This was determined by CLSM optical sectioning. CBM3a, a CBM with specificity toward crystalline cellulose, also labelled most of the cell wall except for the isthmus band (Fig. 1F). CLSM-based optical sectioning through the wall layers revealed CBM3a labelling at the innermost region of the wall that is generally uniform, but interrupted by unlabelled puncta (Fig. 1F). Approximately 10–12 of these puncta were found per square micrometre, matching the number and pattern of the outer wall layer lattice projections observed in the JIM5-labelled cell walls. These unlabelled puncta were interpreted as the shadows of the unlabelled HG of the outer and medial layers embedded in the cellulose domain. Control labelling experiments for the initial immunocytochemical screening included elimination of the primary mAb (Fig. 1G) or CBM3a (Fig. 1H).

### Morphology and growth dynamics of oryzalin-treated *Penium*

*Penium* has a uniform cylindrical morphology consisting of two ‘equal’ sized semi-cells attached at the central isthmus. During most of the cell cycle, the nucleus resides at the isthmus and is flanked by two chloroplasts housed within each semi-cell (Fig. 2A). After 24h of treatment with 280nM oryzalin, noticeable swelling occurred at the isthmus zone (Fig. 2B), which increased further after 36h (Fig. 2C). The nucleus remained in the swollen isthmus and became ensheathed by the chloroplast filling this zone. After 48h, the swelling increased dramatically (Fig. 2D), creating a large spherical central zone within the cell that was sandwiched between the two cylindrical polar zones, suggesting that oryzalin affects the new but not the pre-existing cell wall. Cells did not divide when treated with oryzalin but remained alive for up to 96h, after which the protoplast and cell wall often ruptured at the isthmus. Recovery experiments, involving removal of oryzalin and transfer of the cells to fresh WHM, resulted in a return to the cylindrical morphology. The time taken for recovery was dependent on the time taken for elimination of already internalized oryzalin (Sampathkumar *et al.*, 2011) and, after 12h of recovery, new expansion yielded a narrow, cylindrical morphology arising at the isthmus flanked by the swollen regions that arose during incubation in oryzalin (Fig. 2E). Cells were also able to divide during recovery, yielding products that have narrow cylindrical morphology at the poles (i.e. formed during pre- and post-oryzalin-treatments) and a swollen central region (i.e. formed while incubated in oryzalin; Fig. 2F).

**Fig. 3.**
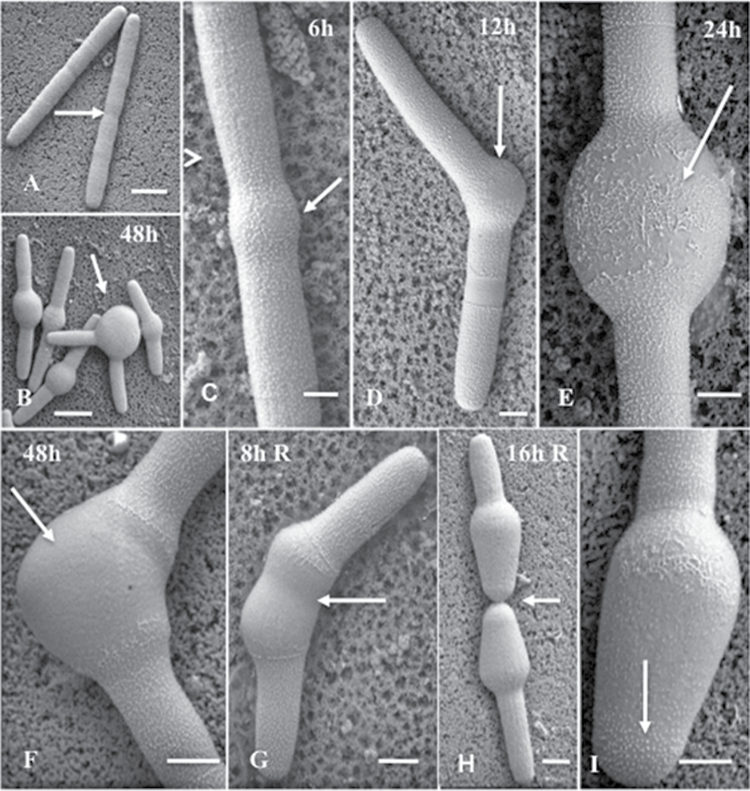
VPSEM imaging of oryzalin-treated cells. (A) Untreated cells. Scale bar=40 μm. (B) Cells treated with oryzalin for 48h showing swollen isthmus regions (arrows). Scale bar=50 μm. (C) Six hours after treatment. Scale bar=11 μm. (D) Twelve hours after treatment. Scale bar=15 μm. (E) Twenty-four hours after treatment, showing a disrupted outer layer HG lattice (arrows). Scale bar=12 μm. (F) After 48h of treatment, little of the outer layer HG lattice is found on the isthmus swelling. Scale bar=12 μm. (G) After 8h of recovery, the cylindrical morphology begins to reform in the isthmus (arrow). Scale bar=15 μm. (H) Cell division after 16h of recovery. Scale bar=30 μm. (I) Magnified view of the expanding polar zone of a daughter cell 24h after recovery showing the outer layer HG lattice reforming in the polar tip (arrow). Scale bar=8 μm.

VPSEM was used to analyse the severe morphological changes to the cell and alterations to the cell wall surface resulting from oryzalin treatment (Fig. 3A, B). More specifically, isthmus-based swelling occurred from 6h to 24h after the treatment (Fig. 3C–E), during which time the HG lattice of the outer wall became disrupted, before ultimately disappearing at ~48h treatment (Fig. 3F). During the recovery experiments, a narrowing of the isthmus region became apparent (Fig. 3G), similar to that observed by LM (Fig. 2E). Cell division reinitiated during recovery (Fig. 3H) and the outer HG lattice reappeared in the growing zone at the polar tip of the expanding daughter cell (Fig. 3I).

### Wall compositional modifications induced by oryzalin treatment

**Fig. 4.**
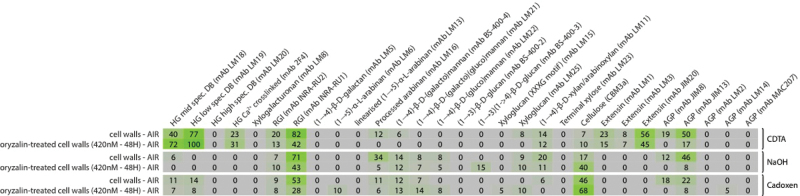
Polysaccharide microarray analysis of the alcohol-insoluble residue (AIR) from untreated *Penium* cells and *Penium* cells treated for 48h with oryzalin. Cell wall polysaccharides were sequentially extracted from the AIR with 50mM CDTA, 4M NaOH, and cadoxen. The solubilized polysaccharides were printed as microarrays and probed with a range of monoclonal antibodies (listed in the top row). The highest mean signal value in the entire data set was set to 100 and all other signals adjusted accordingly. HG, homogalacturonan; RGI, rhamnogalacturonan I; AGP, arabinogalactan protein; CBM, carbohydrate-binding module.

Polysaccharide microarray analysis was performed in order to compare the relative amounts of epitopes of wall polymers in untreated cells with those treated with oryzalin for 48h (Fig. 4). This semi-quantitative technique has been used successfully with several CGA species (Sørensen *et al.*, 2011) and involves sequential extraction of cell wall polysaccharides using CDTA, followed by sodium hydroxide, and cadoxen (diaminoethane and cadmium oxide), prior to spotting onto a nitrocellulose membrane and probing with mAbs or CBMs with specificity for a range of cell wall epitopes. Differences in the mean spot signal intensities were observed after probing with several mAbs, but one particularly striking change was an increase in oryzalin-treated cells of the relative levels of HG epitopes, as recognized by the mAbs LM18 and LM19 which represent new mAbs that label HG epitopes in a similar fashion to JIM5 (Verhertbruggen *et al.*, 2009). Conversely, the relative levels of the RG-I backbone epitope, recognized by the mAbs INRA-RU1 and INRA-RU2, AGP epitopes, recognized by the mAb JIM8, and LM16 and extensin epitopes, recognized by mAbs JIM20 and LM1 all decreased in oryzalin-treated cells. Another notable finding was that the RG-I epitopes were almost as abundant in material extracted with NaOH as in material extracted with CDTA. It should be noted that equal volumes of extract are used for each spot, which allows comparisons between treatments (i.e. with or without oryzalin) but not between extracts (i.e. CDTA, NaOH, and cadoxen) and so the extractability of the epitope-bearing polymers is not considered here. The results of this microarray study were used as a guide for choosing specific targeted polymers for subsequent labelling.

### Immunocytochemical examination of alterations to the HG lattice in oryzalin-treated cells

**Fig. 5.**
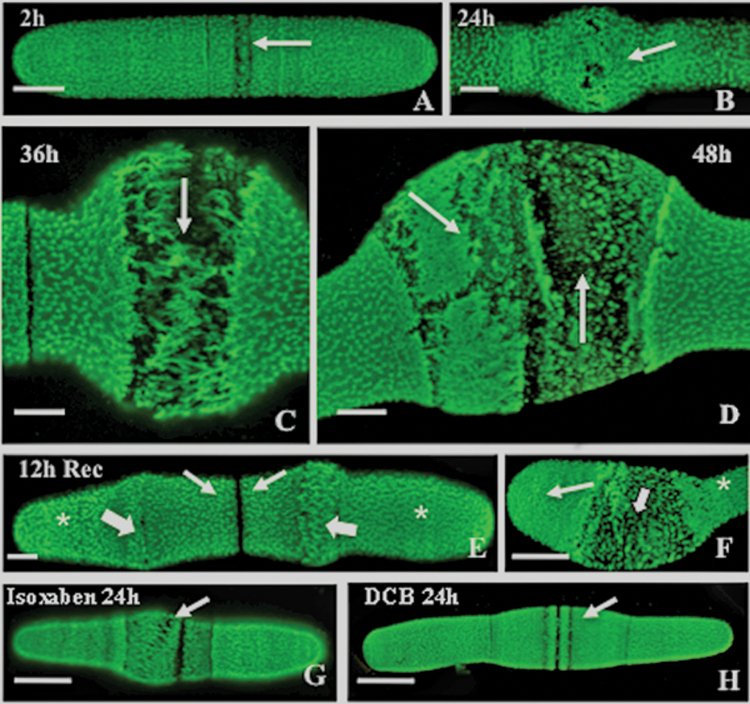
JIM5 (specificity toward relatively low esterified HG) labelling of oryzalin-treated cells. (A) Two hours of treatment showing the beginning of the disruption of the outer wall layer lattice (arrow) at the isthmus band. Scale bar=7.0 μm. (B) Twenty-four hours of treatment showing pronounced isthmus swelling (arrow) and irregular gaps in the outer layer HG lattice at the isthmus. Scale bar=5.0 μm. (C) Thirty-six hours of treatment showing disruption to the HG lattice at the swollen isthmus (arrow). Scale bar=4.0 μm. (D) Forty-eight hours of treatment, showing severe disruption of the HG lattice (arrows). Scale bar=4.0 μm. (E) At 12h after recovery, the typical HG lattice begins to regenerate at the isthmus (small arrows) while the disrupted lattice of the oryzalin-induced swollen zones remains (large arrows). Scale bar=4.0 μm. (F) A recently divided daughter cell, 24h after recovery, initiating a return to the cylindrical shape. The typical HG lattice at the expanding pole (small arrow) is apparent, as is the remnant of the disrupted swollen zone (large arrow) and the original wall of the cell prior to oryzalin treatment (*). Scale bar=15 μm. All images taken with CLSM. (G) Twenty-four hours of treatment with 10 μM isoxaben results in swelling of the isthmus region and disruption of the HG lattice (arrow). Scale bar=15 μm. (H) Twenty-four hours treatment with 0.2 μM DCB results in swelling of the isthmus region and disruption of the HG lattice (arrow). Scale bar=15 μm.

JIM5 was used as a marker to monitor lower DE HG during wall development. This antibody has previously been successfully employed for live cell immunolabelling of *Penium* in the past (Domozych *et al.*, 2009*b*, 2011). Oryzalin treatment (Fig. 5A) initially (~2h) resulted in a narrow region of disruption to the HG lattice of the outer wall layer at the isthmus band (Fig. 5A). After 24h of treatment (Fig. 5B), distinct breaks in the HG lattice were visible and these progressively expanded over 36h and 48h of treatment (Fig. 5C, D), at which point the lattice was severely disrupted. When cells were allowed to recover for 12h, the HG lattice and cylindrical shape reappeared at the isthmus band (Fig. 5E) and at the expanding polar zone of recently divided daughter cells (Fig. 5F). Quantitative analysis of SA coverage by the HG lattice showed that the percentages of cell SA covered by new cell wall in cells treated for 48h and 72h and untreated cells were approximately equal (Table 1). However, in untreated cells, new wall material was found primarily in new cylindrical growth at the isthmus zone whereas in treated cells the majority of new wall material was present in the spherically swollen isthmus. Experiments were carried out to determine if cellulose alteration caused by treatment with the known cellulose synthesis-disrupting agents, isoxaben and DCB, affected the expansion zone and cell shape. When treated with isoxaben (10 μM; Fig. 5G) or DCB (0.2 μM: Fig. 5H), similar swelling of the isthmus was observed. These effects were also reversible by extensive washing of the cells and removal of the disrupting agent.

**Table 1.** Surface area analysis of untreated versus oryzalin-treated cells Triplicate aliquots of 100 cells per calculation were used (±0.3%).

Treatment	Untreated, 48 h	Treated, 48 h	Untreated, 72 h	Treated, 72 h
% SA of new wall versus whole cell SA	63%	62%	90%	90%

### FESEM imaging of HG lattice alteration

**Fig. 6.**
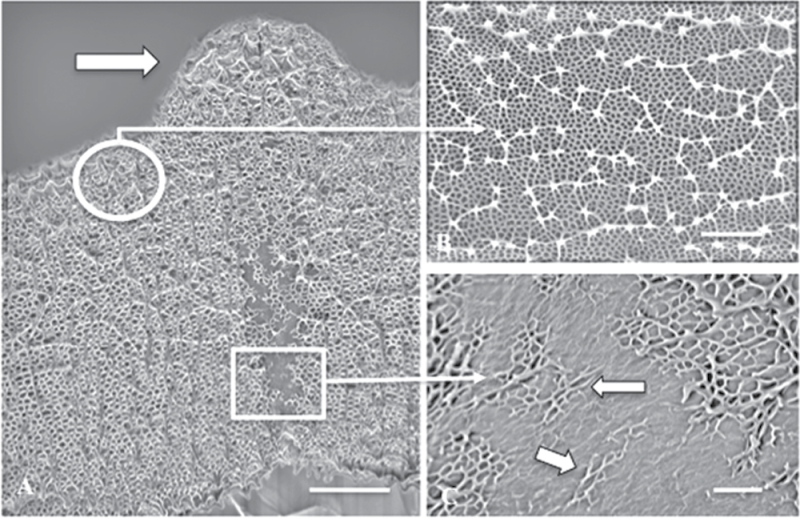
SEM imaging of oryzalin-induced effects on the wall lattice. (A) FESEM image of a cell treated with oryzalin for 24h, highlighting older, unaffected wall (circle) and wall found at the swollen isthmus affected by oryzalin (square). Scale bar=3.5 μm. (B) FESEM image of unaffected wall showing the typical outer HG lattice. Scale bar=1.2 μm. (C) FESEM image of altered wall highlighting the patches of HG in the disorganized lattice (arrows). Scale bar=1.0 μm.

FESEM was employed to provide detailed surface imaging of the cells. During cell swelling at the isthmus band upon 24h treatment with oryzalin, the HG lattice began to tear apart (Fig. 6A). The lattice of the pre-existing cell wall consisted of an inner fibre-based network and outward-extending projections (Fig. 6B). The wall of the swollen isthmus was highlighted by irregular patterns of the HG fibres interspersed with wall regions possessing no lattice (Fig. 6C). These observations corresponded well with JIM5-labelled cells displaying lattice alterations (Fig. 5B–D).

### Effects of oryzalin on enzymatically treated cells

**Fig. 7.**
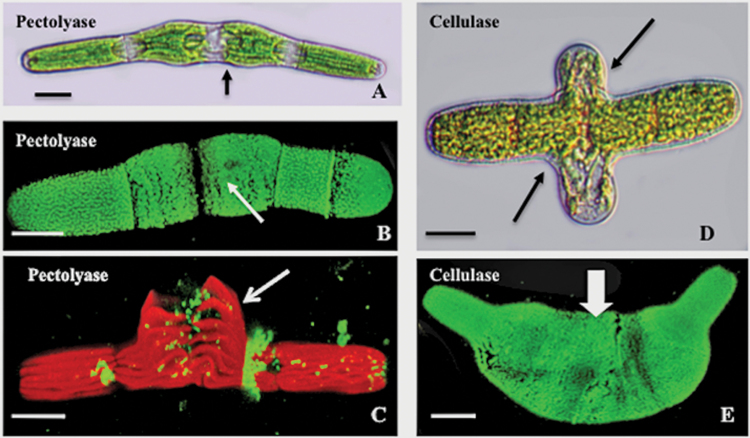
Effects of glycanase pre-treatments before and during oryzalin incubation. (A) The swollen isthmus zone (arrow) of a cell treated with pectate lyase (PL) followed by incubation with oryzalin and PL for 24h. Scale bar=15 μm. DIC image. (B) JIM5 labelling of a cell treated as in (A) showing perturbation of the HG lattice at the swollen isthmus (arrow). CLSM image. Scale bar=7.0 μm. (C) JIM5 labelling of a cell treated as in (A) but with oryzalin/PL for 36h. Little lattice is visible, especially at the swollen isthmus (arrow). Red autofluorescence of the underlying chloroplast is also visible. Scale bar=17 μm. CLSM image. (D) Cell treated with cellulase for 24h and then with oryzalin/cellulase for 24h, resulting in severe swelling at the isthmus zone (arrows). Scale bar=14 μm. DIC image. (E) JIM5 label of a cell treated as in (D) showing altered lattice at the swollen isthmus. Scale bar=13 μm. CLSM image.

In order to elucidate further the effects of oryzalin treatment on the pectin and cellulosic domains, cells were treated with different wall-degrading enzymes prior to oryzalin treatment. When cells were treated with PL for 24h and then incubated for 24h with oryzalin and PL, the isthmus-based swelling was still apparent (Fig. 7A) and the lattice of the outer wall layer, as labelled by JIM5, was disrupted (Fig. 7B). When cells were pre-treated with PL for 48h (2X pre-treatment time), only small strips of lattice remained (Fig. 7C) and cell morphology was similar to that seen when cells were treated with oryzalin alone. However, when cells were pre-treated with cellulase for 24h followed by oryzalin/cellulase treatment, the isthmus-based swellings became highly pronounced (Fig. 7D). The pectin lattice was disrupted (Fig. 7E) and ~20% of these cells ruptured at the isthmus upon pressure of the coverslip. Cells return to normal morphology after recovery via washing (not shown).

### Immunocytochemical analysis of high esterified HG, RG-I, and cellulose during oryzalin treatment

**Fig. 8.**
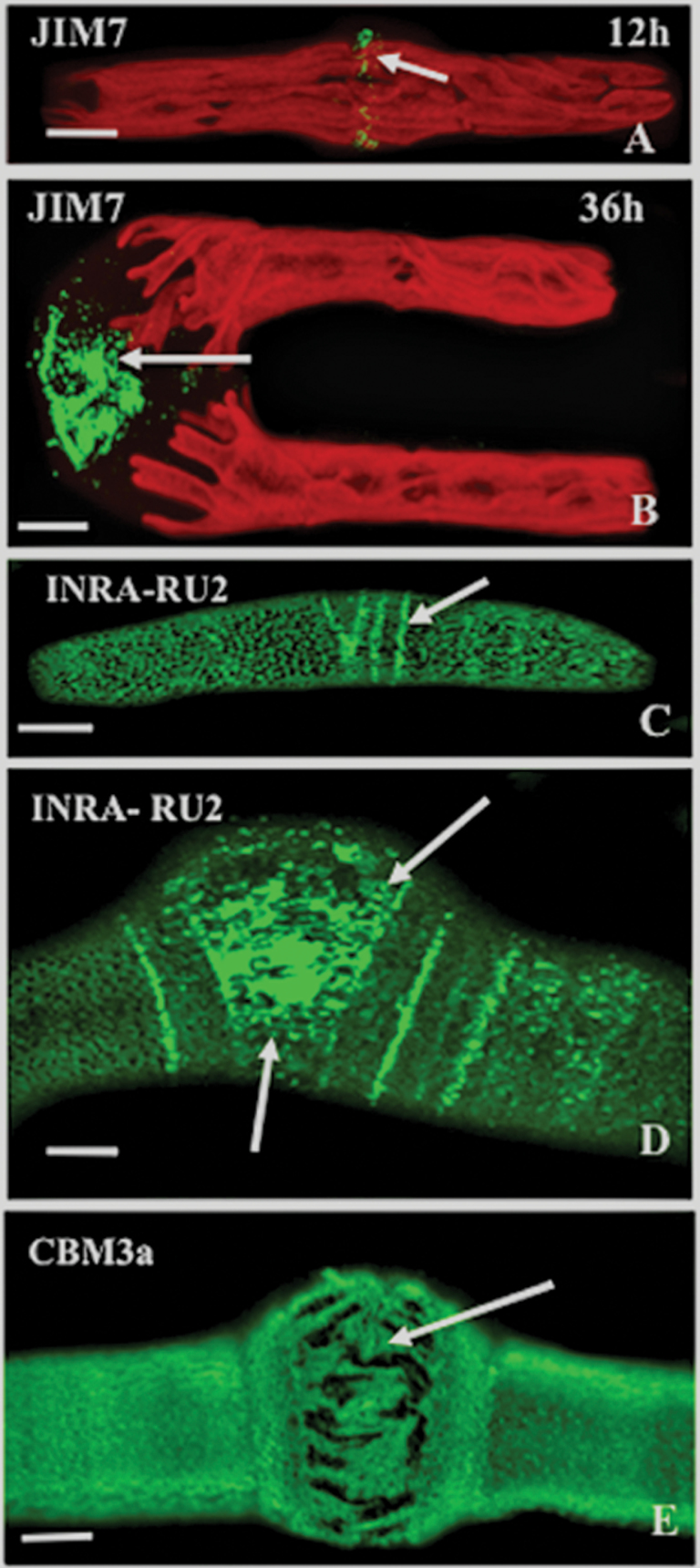
JIM7 (specificity toward relatively highly esterified HG), CBM3a (specificity toward crystalline cellulose), and INRA-RU-2 (specificity toward the backbone of RG-I) labelling of oryzalin-treated cells. (A) The JIM7-labelled band of higher DE HG (arrow) in the swollen isthmus zone after 12h of oryzalin treatment. Chloroplast autofluorescence is also visible. Scale bar=12 μm. (B) Diffuse pattern of JIM7 labelling in the swollen isthmus after 36h (arrow). Scale bar=9.0 μm. (C) INRA-RU2 labelling after 12h showing striations in the isthmus (arrow). Scale bar=10 μm. (D) INRA-RU2 labelling after 36h. Highly irregular labelling occurs in the isthmus zone (arrows). Scale bar=5 μm. (E) CBM3a labelling after oryzalin treatment. The labelling at the isthmus has a shredded appearance (arrow) at 36h. Scale bar=6 μm. All images were taken using CLSM.

Other types of labelling were performed in order to obtain a more complete picture of wall alterations. Labelling of treated cells with JIM7 revealed that oryzalin also affected the distribution of pectins with a higher DE. After 12h of treatment, the typical distribution of label in the narrow isthmus band was observed, even in the swollen isthmus zone (Fig. 8A) but, after 36h, the signal became more diffuse and was irregularly displaced over the central part of the swollen zone (Fig. 8B). Other aspects of the pectin network were disrupted by oryzalin, as evidenced by INRA-RU2 labelling of the RG-I backbone. This showed intensely labelled striations after 12h (Fig. 8C) and then a highly irregular pattern in the swollen isthmus after 36h (Fig. 8D). The spatial distribution of crystalline cellulose, as detected using CBM3a, was similarly perturbed by the oryzalin treatment (Fig. 8E), resulting in a shredded appearance at the swollen isthmus region.

### Cytoskeletal changes induced by oryzalin treatment

**Fig. 9.**
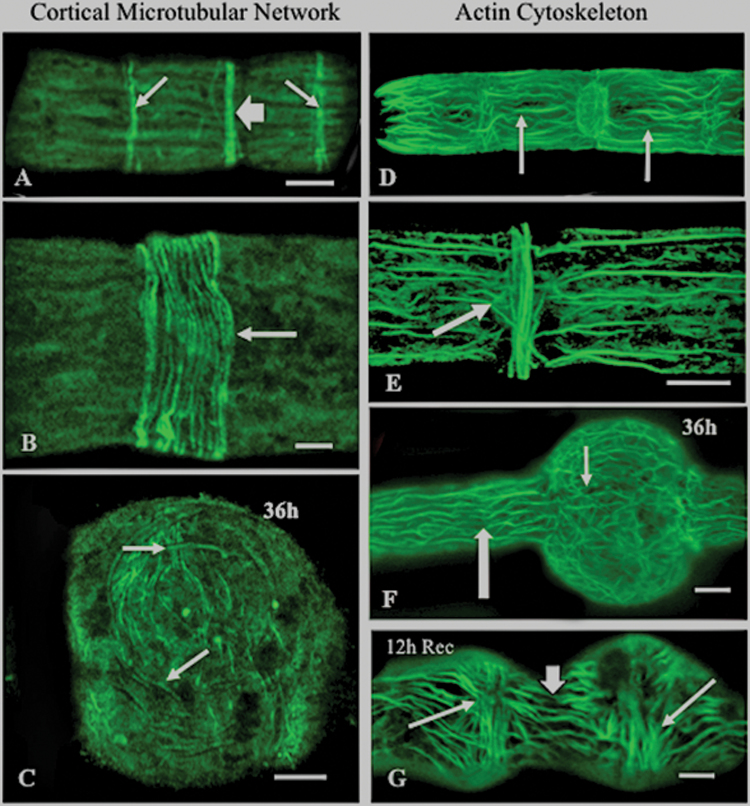
The cytoskeleton and oryzalin-induced alterations. (A) Anti-tubulin labelling of an untreated cell, showing peripheral bands of microtubules (thin arrows) and a denser band at the isthmus zone (thick arrow). Scale bar=8 μm. (B) Magnified view of the microtubule band located in the isthmus (arrow). Scale bar=3 μm. (C) Disorganized microtubule distribution (arrow) in the swollen isthmus after 36h. Scale bar=7 μm. (D) Rhodamine–phalloidin labelling of the actin microfilament network in an untreated cell showing bundles parallel to the long axis of the cell (arrows). Scale bar=4.0 μm. (E) Parts of the cortical microfilament network converge inward near the nucleus situated in the isthmus near the isthmus band (arrow). Scale bar=3 μm. (F) The microfilament network after 36h showing disorganization at the swollen isthmus (arrow). Scale bar=8 μm. (J) Reorganization and reorientation of microfilaments (short arrow) in the newly formed zone after 12h recovery. This zone is surrounded by two swollen zones (arrows) formed during oryzalin treatment. Scale bar=6 μm. All images were taken using CLSM.

Oryzalin has previously been demonstrated to be a potent microtubule-affecting agent in land plants (Hugdahl and Morejohn, 1993; Morrissette *et al.*, 2004). Likewise, cortical microtubule and actin microfilament networks have been shown to be closely associated with cell wall synthesis, secretion, and development. (Mutwil *et al.*, 2008; Paradez *et al.*, 2008). In this study, tubulin immunolabelling and rhodamine–phalloidin labelling of actin cables were used to observe the two cytoskeletal networks in order to elucidate any changes to these cytoskeletal components upon treatment with oryzalin. In untreated cells, the cortical microtubule network was highlighted by distinct rings of microtubules aligned perpendicular to the long axis of the cell (Fig. 9A). The isthmus region contained the largest ring, consisting of a network of 10–20 parallel-aligned microtubules (Fig. 9B). After 36h of oryzalin treatment, the microtubular network became disorganized and no ring was apparent in the swollen isthmus region (Fig. 9C). Upon recovery, the microtubule band of the isthmus region reappeared within 4h (data not shown). The actin microfilament network of *Penium* consists of parallel arrays of microfilament bundles in the subplasma membrane cortical region running parallel to the longitudinal axis (Fig. 9D). At the isthmus zone, parts of these microfilament bundles converged inward to form a ring at the same location as the microtubular band and corresponding to the JIM7-labelled region (Fig. 9E). After 36h of oryzalin treatment, the isthmus-based microfilament band became highly disorganized (Fig. 9F) but the parallel alignment of microfilament bundles in the unaltered regions of the cell remained. After 12h of recovery, the normal distribution of microfilaments returned (Fig. 9G).

### Ultrastructural effects of oryzalin treatment

**Fig. 10.**
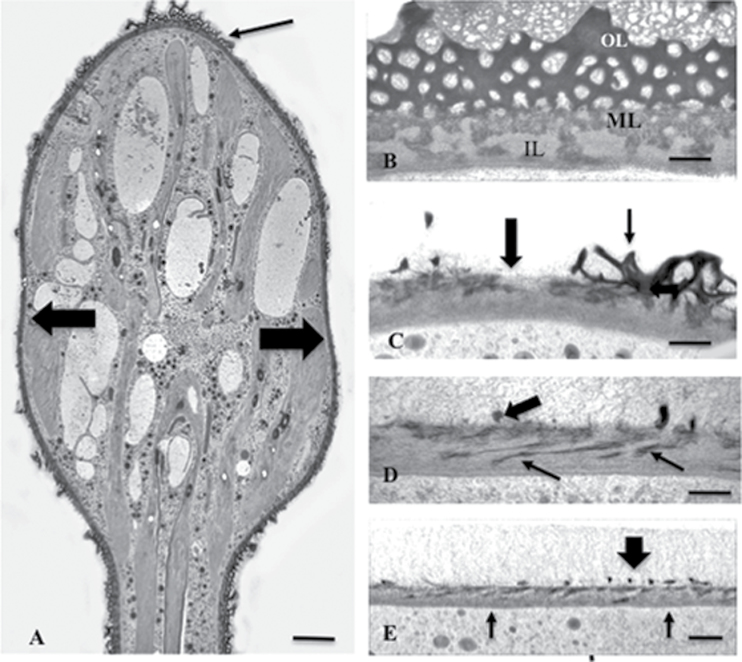
Cell wall ultrastructural changes during oryzalin treatment. (A) Cell treated with oryzalin for 36h showing thinner inner layers and absence of outer layer projections (large arrows). The wall of unaffected zones is also apparent (thin arrow). Scale bar=8.5 μm. (B) The three cell wall layers of non-isthmus regions: an outer layer (OL) consisting of the HG lattice, an inner layer (IL) containing cellulose, and an interface medial layer (ML) with fibrils from the OL HG embedded in the IL. Scale bar=400nm. (C) Cell treated for 24h showing the HG lattice and other wall layers at the edge of the altered cell wall of the swollen isthmus region. The affected region retains the medial layer (thin arrows) but only remnants of the HG lattice (thick arrow). Scale bar=250nm. (D) Cell treated for 36h showing elongated striations of the medial layer within the inner layer (thin arrows) and only a few remnants of the outer layer lattice (thick arrow). Scale bar=250nm. (D) Cell treated for 48h showing few remnants of the HG lattice (thick arrow), reduction of the medial layer, and a thinner inner cellulose layer (thin arrows). Scale bar=600nm. All images were taken using TEM.

The effects of oryzalin on cell wall ultrastructure were assessed using TEM. After 48h of treatment, noticeable alterations to the wall were observed (Fig. 10A). In addition to alterations in the HG lattice, the wall was thinner, and little, if any, lattice was apparent. The *Penium* cell wall consists of three layers, an outer layer containing the HG lattice, an inner fibrous layer of cellulose, and a middle ‘interface’ layer where the HG of the outer layer embeds in the cellulose (Fig. 10B). Treatment of cells with oryzalin for 24h (Fig. 10C) resulted in a notable disruption of the wall architecture with a sharp interface between altered and unaltered regions of the wall. All three wall layers were present in the region formed before oryzalin treatment. However, in wall formed during oryzalin treatment (i.e. the swollen zone), little of the HG lattice remained. After longer treatments (36h), the medial layer appeared as multiple linear ‘streaks’ positioned nearly perpendicular to the long axis of the wall (Fig. 10D). These micrographs were taken from sections of cells embedded in plastic sheets to enable observation of their longitudinal wall profiles. After 48h (Fig. 10E), the cell wall of the swollen region became notably thinner and contained remnants of the medial layer components located at the outermost region of the inner layer. In a comparison of 50 micrographs of the cell walls of treated and untreated cells, oryzalin treatment resulted in a decrease of 25% (±4%) of the inner/medial wall layer thickness.

## Discussion

### Oryzalin induces a loss of wall biomechanical strength at the primary site of wall deposition

The cell wall of *Penium* consists of two major domains that are arranged in three recognizable layers. One domain consists of HG (Domozych *et al.*, 2007; Sørensen *et al.*, 2011) that binds with Ca^2+^ to form the distinctive lattice that constitutes the outer layer of the wall. The second domain is cellulose based (Domozych *et al.*, 2011) and makes up the inner cell wall layer. Aggregates of HG fibrils emerging from the base of the outer layer embed in the microfibrillar infrastructure of the inner cellulose-rich layer and form the medial layer. This layer contains both HG and RG-I, and represents the zone where pectin and cellulose physically intersect. This architectural design of the cell wall supports the elongate cylindrical shape of the cell and resists the pressures of internal turgor. Treatment of *Penium* with oryzalin compromises this cylindrical design and causes distinct swelling at the isthmus zone. This swelling is accompanied by significant alterations to both wall domains and the overall structural architecture of the wall. The isthmus is the site of the isthmus band during pre-division expansion where HG is secreted and incorporated into the wall, where cellulose microfibrils are synthesized, and where the pectic and cellulosic domains most probably become interconnected (also see Domozych *et al.*, 2009*b*). Consequently, the developing cell wall at the isthmus band is more elastic than at other parts of the cell and is more susceptible to the pressure of internal turgor if its structural integrity is compromised. This would explain why oryzalin-induced swelling occurs here. Alternatively, oryzalin does not affect pre-existing wall, suggesting that the mature wall is not significantly remodelled after it forms, or that any post-synthesis remodelling is not affected by application of oryzalin.

The mechanism of highly focused wall expansion and the oryzalin-induced swelling at the isthmus band in *Penium* have some notable similarities to that observed in other anisotropically growing plant cells. For example, in expanding pollen tubes, the focal point of wall expansion is also a narrow band, specifically the apical zone located at the tube tip (Geitmann and Steer, 2006; Geitmann and Ortega, 2009; Aouer *et al*., 2010; Dardelle *et al.*, 2010; Fayant *et al.*, 2010; Geitmann, 2010; Cai *et al.* 2011). Oryzalin treatment also causes swelling at this apical tip (Anderhag *et al.*, 2000). In the pollen tube apex, high DE HG secretion and callose/cellulose synthesis produce an elastic wall zone capable of regulating turgor-driven expansion. Immediately beyond the apex, pectin methylesterase (PME) remodelling of the HG followed by Ca^2+^ cross-linking creates a rigid gel which strengthens the wall that will surround the long tube shank. In *Penium*, the isthmus band is functionally equivalent to the expanding apical tip of the pollen tube; that is, where HG secretion/modelling and cellulose microfibril synthesis actively occur. However, the *Penium* wall synthesis mechanism differs from that of pollen tubes in that although there is a single wall expansion zone (the isthmus band), wall expansion is bi-directional. This predicates the presence of a currently described mechanism that allows for both PME processing of secreted HG and displacement of this HG toward both poles of the cell.

### Oryzalin disrupts *Penium* microtubular dynamics and wall deposition but not cell expansion

In previous studies, oryzalin has been shown to affect microtubule dynamics in plants and some protists directly by sequestering tubulin dimers (Hugdahl and Morejohn, 1993; Morrissette *et al.*, 2004). In land plants, this leads to changes in cell wall infrastructure and subsequent cell swelling (Nakamura *et al.*, 2004; Bannigan *et al.*, 2006; Paradez *et al.*, 2006; Corson *et al.*, 2009) similar to that observed in this study. In *Penium*, it was shown that parallel bands of cortical microtubules aligned perpendicular to the cell’s longitudinal axis are found in the central region of the cell, the largest and most prominent of which resides at the isthmus band. More importantly, this microtubule band was dramatically altered during oryzalin treatment, resulting in a random display of microtubules dispersed throughout the cytoplasm of the isthmus. This corresponded to alterations to the cell wall and the swelling at the isthmus region. What might be the link between the cortical microtubular cytoskeleton and wall expansion dynamics occurring at the isthmus band? Throughout the past half-century of cell wall research, close associations of cortical microtubules with cellulose microfibril orientation have been noted in many plant cells (Smith and Oppenheimer, 2005; Mutwil *et al.*, 2008; Lloyd and Chan, 2008; Anderson *et al.*, 2010; Chan *et al.*, 2010; Endler and Persson, 2011). Recently, live cell imaging using fluorescent protein fusions with cellulose-synthesizing enzymes (e.g. cellulose synthase, or CesA complexes) has further demonstrated the dynamic interaction between the cellulose synthetic machinery residing on the plasma membrane and the underlying layer of cortical microtubules (Paradez *et al.*, 2006). It is widely believed that cortical microtubules serve as guides that direct the movement of cellulose synthase complexes on the plasma membrane and, in turn, the production of cellulose microfibrils in specific orientations in the cell wall. According to this model, perturbation of the cortical microtubular network by an agent such as oryzalin would affect the synthesis of the cellulose microfibrillar network. The results of this study also suggest that alteration of the cortical microtubule network by oryzalin in the active wall expansion zone, the isthmus band, directly affects both the cellulose synthesis machinery and wall microarchitecture. For example, TEM imaging demonstrated that the cellulose-based inner wall layer was reduced in thickness by 25% at the oryzalin-induced swollen zones. It is possible that the microtubule disruption at the isthmus band slows or alters cellulose microfibril synthesis, yielding a thin cellulosic layer. Turgor pressure at this thin zone would then cause deformation of cell shape. The cellulosic framework here would still be sufficient to keep the cell from bursting but would be unable to maintain the narrow cylindrical shape at the isthmus (i.e. swelling occurs). It is also possible that oryzalin-induced alteration of the cellulose synthesis machinery causes an increased stretching in the cellulosic layer (i.e. increased sliding of microfibrils) which then contributes to the thinning of the inner layer and subsequent perturbation of the HG lattice. The link of oryzalin treatment to cellulose domain disruption is further strengthened by observations from this study whereby cellulose-affecting agents (e.g. isoxaben or DCB) also cause swelling at the isthmus. Interestingly, the present study also showed that in oryzalin-treated cells, the percentage of surface area covered by new wall material in relation to the whole cell was approximately the same as in untreated cells. This suggests that while structural changes occurred in the wall following oryzalin treatment, the rate of wall expansion is not noticeably altered. The geometry of expansion changes from linear (cylinder) to spherical (swollen isthmus) but not the amount of cellular expansion.

### Evidence for coordinated deposition and interaction of the pectin and cellulose cell domains

**Fig. 11.**
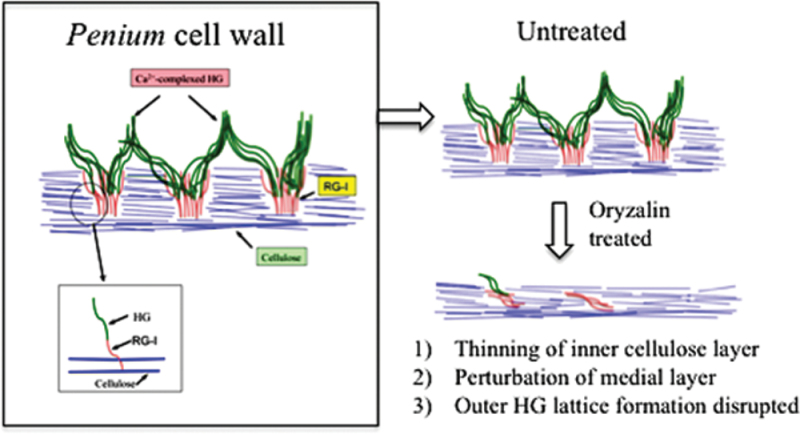
Schematic diagram of the structural changes that occur to the cell wall during oryzalin treatment. The cell consists of an inner layer of cellulose, an outer layer of HG that forms the distinct lattice, and a medial layer that consists of RG-I and HG. It is at this layer where the pectin and cellulose are connected. Upon oryzalin treatment, cellulose synthesis is altered, resulting in a thinner cellulosic layer. Subsequently, this perturbs the formation of the RG-I-containing medial layer that in turn disrupts the formation of the HG lattice. (This figure is available in colour at *JXB* online.)

The thinning of the cellulosic inner layer during oryzalin treatment also results in distinct alterations to the pectin domain of both the medial and outer layers. Proper formation of the cellulosic layer probably serves as the framework for the deposition and anchoring of the HG-based outer layer. When formation of this cellulosic layer becomes compromised by oryzalin, alteration of the HG lattice also occurs (Fig. 11). This result exemplifies a complex structural interaction between two polymer domains that must be developmentally coordinated and adds to the growing evidence supporting pectin–cellulose interactions (Zykwinska *et al*., 2005, 2007; Peaucelle *et al.*, 2012). In *Penium*, the identification of RGI in the medial layer suggests that this polymer may also be involved in this interaction, although its relative abundance appears to be relatively low compared with that of land plants (Sørensen *et al.*, 2011).

Previous research has shown that pectins and pectin-modulating enzymes such as PME (Mohnen, 2008; Bosch and Hepler, 2005; Tian *et al.*, 2006), as well as the cellulose synthesis machinery (e.g. CesA; Mutwil *et al.*, 2008; Petrasek and Schwarzerova, 2009; Endler and Persson, 2011) are produced in the Golgi apparatus (GA) and transported by GA-derived vesicles via actin-mediated movement to the cell surface (Bove *et al.*, 2008; Cheung *et al.*, 2008; Daher and Geitmann 2011). In this study, rhodamine–phalloidin labelling revealed an extensive network of actin microfilaments found in the peripheral cytoplasm where it aligned parallel to the longitudinal axis of the cell. Some of this network converges inward at the nucleus that resides in the same location as the isthmus band. *Penium*, like most desmids, also displays active cytoplasmic streaming in the peripheral cytoplasm that is directed along the longitudinal axis of the cell. These observations led to the presumption that actin-mediated cytoplasmic streaming is also a major mechanism for transporting Golgi-derived secretory vesicles in *Penium*, including those carrying wall polymers or polymer-biosynthetic/modulating enzymes to a wall expansion site at the cell surface, namely the isthmus band. It was also shown that oryzalin treatment results in a localized disorganization of the actin microfilament network in the affected region of the cell, the swollen isthmus. These observations led to the belief that oryzalin-induced alterations in cell wall development in *Penium* may be due to perturbation of the actin network that subsequently disables or significantly alters delivery of CesA complexes being transported from the GA to the plasma membrane at the isthmus band. This would, in turn, affect cellulose microfibril production at the isthmus band and initiate a cascade whereby the wall at this band would no longer maintain its normal tensile strength which is responsible for restricting turgor-driven pressure, thus leading to the observed swelling. The alteration of the actin network might also directly affect pectin secretion at this wall expansion site (e.g. compromised delivery of HG-carrying vesicles). Though oryzalin is a microtubule poison, it has been previously demonstrated that there is a close association of microtubules with microfilaments (Szymanski and Cosgrove, 2009; Sampathkumar *et al.*, 2011). In *Penium*, the cortical cytoplasm is highlighted by both microtubule and microfilament bands. If the microtubule network is disrupted by oryzalin, subsequent disruption of the actin network may also occur, leading to the aforementioned alterations in wall development and structure.

### The pectin and cellulose domains: physically interacting but distinct functions?

This study has shown that oryzalin affects the architecture of both the cellulose and pectin domains of the cell wall and manifests in a major change to cell shape. These observations led to the question of which cell wall polymer and/or domain is primarily responsible for maintaining the structural integrity of the wall at the isthmus band and the cylindrical morphology of the cell. First, in untreated cells, the cell wall of the isthmus band consists primarily of the cellulose-rich inner layer (i.e. no HG lattice) and the typical cylindrical cell shape is maintained here. In oryzalin-treated cells, the cellulose layer at the isthmus band thinned, and swelling of the isthmus region occurred. Secondly, in cells pre-treated with cellulase and then treated with oryzalin and cellulase, the swelling at the isthmus zone became even more pronounced and, in some cases, leads to wall rupture. These observations indicate that the cellulosic inner layer is most important in resisting inner turgor pressure driving expansion and in maintaining the cylindrical cell shape. If this cellulose-based infrastructure is compromised, as it is with oryzalin treatment, cell wall integrity and its tensile resistance to turgor-driven pressure are also compromised. This observation closely corresponds to other studies that show that if cellulose infrastructure is altered at an expansion site, the tensile resistance of the cell wall and/or cell shape may be severely altered (Aouer *et al*., 2009).

What then is the role of the HG, and particularly the prominent HG lattice that covers most of the cell surface, in the structural mechanics of the cell wall? First, the present polysaccharide microarray analysis showed that levels of HG epitopes notably increased following oryzalin treatment. It may be the case that if, as suggested, HG is required for maintaining wall integrity in expanding *Penium* cells then the indirect disruption of cellulose synthesis and/or orientation caused by oryzalin treatment led to a compensatory increase in HG synthesis and/or deposition, as has been suggested to occur in land plants (Burton *et al.*, 2000; Bischoff *et al*., 2008). If so, this is reminiscent of the effect of the cellulose inhibitor isoxaben on cell cultures, which causes a disruption of the cellulose crystallinity and a, presumably compensatory, increase in HG (Bernal *et al.*, 2007). In this regard, it is probably significant that the polysaccharide microarray analysis showed a change in the binding of 2F4, which suggests that the effect of oryzalin is not just to induce production of HG *per se*, but rather the production of HG with sufficient contiguous non-methyl-esterified GalA residues to participate in the structurally important process of Ca^2+^ cross-linking. It should be noted that oryzalin may also exert a direct effect on the activity of PME, similar to its action on other wall enzymes (Vissenberg *et al.*, 2005), which, in turn, affects its remodelling of secreted HG. However, while oryzalin treatment causes significant alteration to the HG production levels and the lattice infrastructure is disrupted at the swollen isthmus region, comparable experiments with PL pre-treatment followed by oryzalin treatment do not result in further cell swelling or rupture, as observed with cellulase pre-treatment (Fig. 7). This indicates that the HG is not primarily responsible for maintaining the structural integrity of the wall or cell shape at the isthmus band.

While further work is needed to resolve the role of the HG, it is suggested that the HG lattice may represent a network of reinforcing struts that are needed to support the large expanse of the elongate cylindrical shape of *Penium*. Struts are mechanical devices often organized in regular networks that are embedded in the external edifice of a structure, functioning to reinforce the integrity of structures that have large longitudinal axes (e.g. cylinders). While not serving as the main structural framework, they nonetheless help maintain elongate structures. Further biomechanical studies will be needed to confirm the role of the HG lattice and elucidate the tensile strength of the cellulose domain. It is also possible that the HG lattice does not affect wall rigidity but may function in cell adhesion. An interesting area of future research will be to determine whether these domains, as well as other wall components such as RG-I, consistently show common organizations and functions in the walls of CGA and land plants.

## Abbreviations:

AGP: arabinogalactan protein
CBM: carbohydrate-binding module
CLSM: confocal laser scanning microscopy
DCB: dichloronitrobenzile
DE: degree of esterification
DIC-LM: differential interference contrast light microscopy
GA: Golgi apparatus
HG: homogalacturonan
LM: light microscopy
mAb: monoclonal antibody
PL: pectate lyase
RG-I: rhamnogalacturonan I
VPSEM: variable pressure scanning electron microscopy
WHM: Woods Hole medium.
